# Triple‐Level Content‐Addressable Vertical NAND Flash Memory

**DOI:** 10.1002/advs.77390

**Published:** 2026-08-24

**Authors:** Sung‐Ho Park, Jiseong Im, Yeongheon Yang, Ryun‐Han Koo, Jonghyun Ko, Mingyun Oh, Dongbeen Shin, Gyuweon Jung, Jong‐Ho Lee

**Affiliations:** ^1^ Department of Electrical and Computer Engineering and Inter‐University Semiconductor Research Center Seoul National University Seoul Republic of Korea; ^2^ Research and Development Division SK Hynix Inc. Icheon Republic of Korea

**Keywords:** content‐addressable memory, few‐shot learning, V‐NAND flash memory

## Abstract

Content‐addressable memory (CAM) enables massively parallel search by directly comparing input data with stored entries, offering a powerful paradigm for accelerating data‐intensive tasks. Here, we present a triple‐level content‐addressable vertical NAND flash memory (V‐NAND TLCAM) that exploits the eight threshold‐voltage states of commercial triple‐level‐cell (TLC) V‐NAND flash devices to achieve compact, high‐throughput, and energy‐efficient in‐memory search. A parity‐split (PS) scheme, in which odd and even word‐lines are accessed separately, enhances the sensing margin and mitigates worst‐case errors while preserving storage density and supporting don't‐care matching. Measurements on >100‐layer TLC V‐NAND flash memory confirm stable operation with up to a 24× improvement in read‐operation throughput and a 54.4× reduction in the estimated read energy compared with conventional readout. Furthermore, few‐shot learning simulations on the Omniglot dataset demonstrate that the proposed TLCAM maintains over 90% accuracy in 5‐shot learning, approaching GPU‐based cosine similarity baselines. These results highlight V‐NAND TLCAM as a scalable and energy‐efficient hardware platform for bridging device‐level innovations with practical machine learning applications.

## Graphical abstract

Commercial triple‐level‐cell V‐NAND flash memory is transformed into a compact content‐addressable memory by directly exploiting its eight threshold‐voltage states. A parity‐split search scheme preserves storage density while enabling reliable multi‐level and don't‐care matching. Parallel word‐line search delivers up to 24‐fold higher throughput and 54.4‐fold lower read energy, while supporting accurate few‐shot learning.

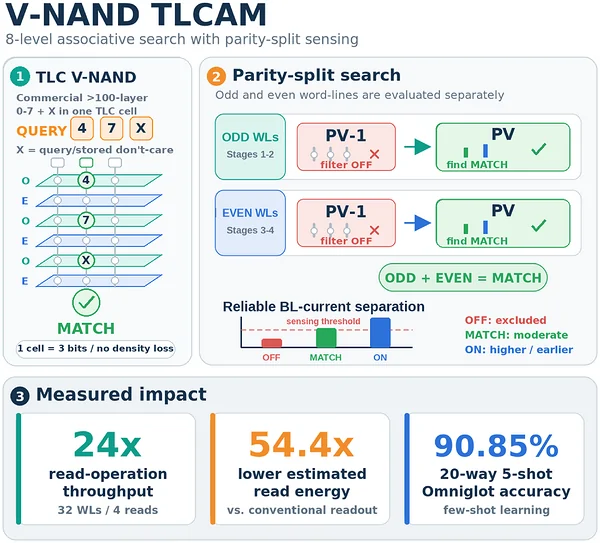

## 1 Introduction

Content‐addressable memory (CAM) is widely recognized as a fundamental computing primitive for accelerating data‐intensive operations [1, 2, 3, 4, 5]. Unlike conventional random‐access memory (RAM), which retrieves information by a physical address, CAM performs associative search by comparing an input query against all stored entries simultaneously [1]. This highly parallel nature enables CAM to complete search operations in a single cycle, making it especially valuable for tasks that require rapid lookup or pattern matching. Over the past decades, CAMs have been integrated into numerous high‐performance systems ranging from high‐speed networking—such as IP address lookup, packet classification, and routing tables–‐to database indexing, security filtering, and real‐time pattern recognition [4]. The extension to ternary CAMs (TCAMs), which add a third “don't‐care (X)” state in addition to binary “0” and “1,” has further expanded the applicability of CAMs by enabling wildcard matches that allow for flexible and compressed representations of rules and patterns [2, 5]. As modern applications continue to generate massive amounts of data, the need for scalable, energy‐efficient, and high‐throughput CAM architectures has become increasingly critical.

At the same time, in‐memory computing (IMC) has emerged as a promising paradigm to overcome the limitations of von Neumann architectures, where frequent data transfers between memory and processors impose severe latency and energy penalties [6, 7, 8, 9]. CAM is an especially compelling form of IMC because each memory cell intrinsically functions as a comparator, enabling computation and storage to be co‐located [3]. Consequently, CAM arrays are capable of executing large numbers of comparisons in parallel with minimal data movement. However, conventional CMOS‐based CAM designs built on static random access memory (SRAM) suffer from low density and significant static power consumption, restricting their scalability to the very large array sizes demanded by artificial intelligence (AI), machine learning, and big‐data applications [2, 3, 10, 11]. This has motivated exploration of CAM implementations using memory technologies such as phase‐change memory (PCM), resistive RAM (ReRAM), magnetoresistive RAM (MRAM), and ferroelectric FETs (FeFETs) [5, 12]. These approaches have demonstrated promising energy and density characteristics, but most remain at the proof‐of‐concept stage and face challenges in manufacturability and large‐scale deployment [10, 13].

In contrast, vertical NAND (V‐NAND) flash memory is already the dominant non‐volatile memory in commercial markets, shipping at multi‐terabit densities [14, 15, 16]. The three‐dimensional stacking of word‐lines (WLs) in V‐NAND flash memory enables extremely high integration density, scalability to hundreds of layers, and competitive cost per bit. Furthermore, V‐NAND flash memory has benefited from decades of process optimization, ensuring robust device characteristics, long retention, and high reliability [15, 16, 17]. These attributes make V‐NAND flash memory an especially attractive candidate for repurposing as a computing substrate [18, 19, 20]. By exploiting its vertical architecture, it becomes possible to align CAM search operations with the stacked WLs, thereby realizing compact and massively parallel search functionality within a platform that is already manufactured at global scale. Such an approach not only leverages a mature technology node but also lowers barriers for near‐term commercialization of in‐memory search engines.

In this work, we introduce a triple‐level content‐addressable V‐NAND flash memory (V‐NAND TLCAM) that directly utilizes the multi‐level states of triple‐level‐cell (TLC) flash cells to implement associative search. Unlike binary or ternary CAMs that operate with two or three logic states, the TLC device offers eight distinct threshold‐voltage levels, thereby enabling richer data representation and enhanced search flexibility. This multi‐level capability provides a natural foundation for implementing not only exact match and “don't‐care” operations but also approximate matching and similarity scoring.

Figure 1 compares the conventional TCAM with the proposed triple‐level CAM (TLCAM). While TCAM supports binary 0/1 values and a don't‐care state, the TLCAM can exploit eight levels (0–7) together with don't‐care, thereby allowing more compact representation of search data and higher flexibility in associative matching. This expanded state space directly reflects the capability of TLC flash devices and highlights the key distinction of our approach.

**FIGURE 1:**
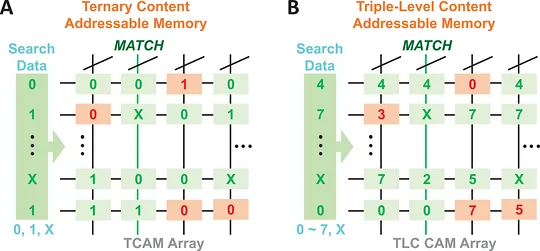
Comparison between conventional TCAM and the proposed V‐NAND TLCAM. Schematic illustration comparing (A) ternary CAM (TCAM), which supports binary 0/1 and a don't‐care (X) state, with (B) the proposed triple‐level CAM (TLCAM) that exploits eight threshold‐voltage levels (0–7) in TLC V‐NAND flash memory together with don't‐care.

To ensure robust functionality despite device variations and worst‐case scenarios, we propose a parity‐split (PS) search scheme in which odd and even WLs are accessed separately, improving the sensing margin and enabling reliable discrimination between matched and mismatched entries. Importantly, this scheme preserves the inherent storage density of V‐NAND flash memory while achieving high search throughput aligned with the vertical structure. Experimental validation using commercial TLC V‐NAND devices confirms stable operation across various search conditions, with up to a 24× improvement in search speed compared to conventional cell‐by‐cell search methods. The energy required per search is also reduced by up to a 54×, since entire WL groups can be processed in parallel rather than sequentially traversing each cell.

Beyond demonstrating device‐ and array‐level operation, we explore the system‐level implications of V‐NAND TLCAM in machine learning tasks, particularly few‐shot learning (FSL). FSL is a challenging paradigm in which a model must classify novel categories using only a handful of labeled examples [5, 21, 22]. Nearest‐neighbor‐based classifiers are well suited for FSL because they can categorize new samples by comparing them directly to stored exemplars. Here, CAM architectures provide a natural hardware accelerator: each query vector can be compared in parallel with all stored prototypes, and similarity can be computed efficiently in‐memory. By leveraging the multi‐level search capability of our V‐NAND TLCAM, we show through simulations that classification accuracy on the benchmark dataset remains competitive with software baselines, while search operations are performed with greatly reduced latency and energy compared to conventional read‐based search within V‐NAND flash memory. This highlights the practicality of the proposed design as a pathway toward efficient associative search in AI workloads, without relying on external accelerators such as graphics processing units (GPUs).

## 2 Results

### 2.1 Architecture of V‐NAND Flash Memory and Prior CAM Approaches

Figure 2A illustrates the schematic structure of a commercial V‐NAND flash memory array and its adaptation to CAM operations. A V‐NAND string is formed by the combination of a bit‐line (BL) and a drain‐select‐line (DSL), with WLs stacked vertically along the channel [23] (Note S1) [24, 25, 26]. Unlike the BL and the DSL, the source‐select‐line (SSL) and the source‐line (SL) are merged into a single structure to reduce resistance [19]. Each BL is connected to a sense‐amplifier (SA), which detects the BL current (*I*_BL_) flowing through the string during read or search operations. Figure 2B illustrates a cross‐sectional view of a single V‐NAND string. During the program operation, electrons tunnel from the channel into the charge‐trap nitride for storage.

**FIGURE 2:**
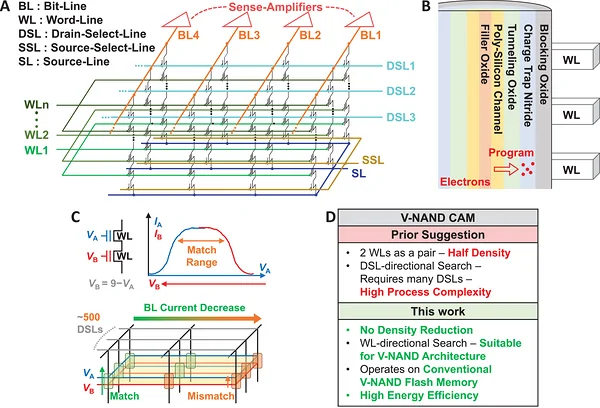
Architecture of V‐NAND flash memory and prior CAM approaches. (A) Schematic structure of a V‐NAND flash memory. (B) Schematic cross‐sectional view of a V‐NAND string. (C) Prior V‐NAND flash‐based CAM design using complementary voltages on adjacent WLs [13, 27]. (D) Comparison between prior DSL‐directional search and the proposed WL‐directional TLCAM.

In reported approaches [13, 27], complementary voltages were applied to adjacent WLs to define the upper and lower bounds of a stored data range, as shown in Figure 2C. When a search input was applied, a high *I*_BL_ was generated if the query fell within this range. If all cells in a string satisfied the search condition, the *I*_BL_ reached its maximum level, enabling the identification of a match event. This concept demonstrated the feasibility of flash‐based CAMs and highlighted the potential of leveraging high‐density NAND arrays for parallel search operations.

Despite its novelty, this scheme faces limitations when applied to commercial‐scale V‐NAND. First, storage density is reduced by half, since two WLs are required to encode each data entry. Second, the search operation is performed along the DSL direction, meaning that the utility of the approach increases with the number of DSLs. However, state‐of‐the‐art V‐NAND typically includes fewer than ten DSLs per block, since increasing the number of DSLs significantly complicates the fabrication process, while the proposed methods often assume hundreds of DSLs to realize large‐scale parallelism. This mismatch leads to significant fabrication complexity and makes practical implementation challenging. Finally, because all non‐searched WLs must be biased with a pass voltage, the DSL‐directional search suffers from high energy overhead, particularly as the number of WLs scales upward. Moreover, since all V‐NAND strings are selected during each search operation, current flows through every string in the block, further exacerbating the overall energy consumption.

Taken together, these prior studies underscore both the promise and the limitations of flash‐based CAMs. They demonstrated that CAM functionality can indeed be realized in V‐NAND, but they also highlighted the trade‐offs between storage density, scalability, and energy efficiency. Motivated by these challenges, our work proposes a fundamentally different approach in which the CAM operation is aligned with the vertical WL direction rather than the DSL direction. Figure 2D presents a comparison between the prior work and the proposed V‐NAND TLCAM. By exploiting the inherent stacking of WLs, our design preserves the full storage density of the array while enabling fast, low‐energy, and scalable search operations.

### 2.2 Operation Method of the Proposed V‐NAND TLCAM

Figure 3 illustrates the operating method of the proposed V‐NAND TLCAM, with detailed views of the cell‐level mechanism (Figure 3A), the sensing principle (Figure 3B), and the array‐level search procedure (Figure 3C). The method builds upon the intrinsic threshold‐voltage (*V*_th_) states of TLC V‐NAND cells, enabling associative search without altering the underlying storage density.

**FIGURE 3:**
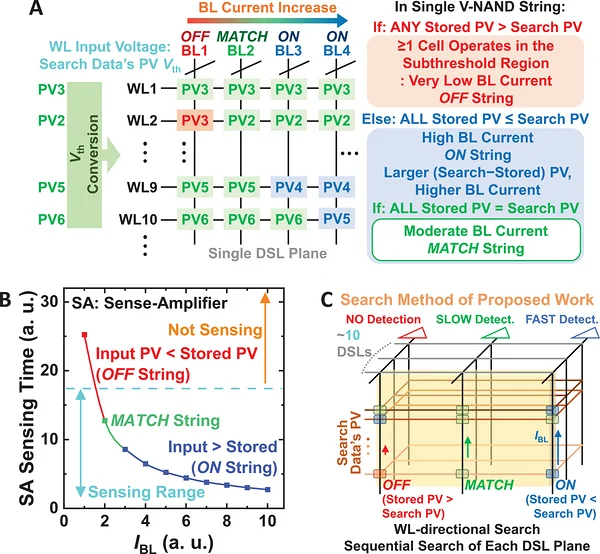
Operation method of the proposed V‐NAND TLCAM. (A) Cell‐level mechanism: WLs biased at the search PV produce BL currents depending on stored PV values, classified into *OFF*, *MATCH*, and *ON* strings. (B) Sensing principle: *OFF* strings generate negligible current and fall outside the sense‐amplifier sensing window; *MATCH* strings are sensed last, while *ON* strings are detected earlier due to larger BL currents. (C) Array‐level search procedure: WL‐directional search across each DSL plane in sequence, contrasting with prior DSL‐based methods.

As shown in Figure 3A, each WL is biased at a voltage corresponding to the program‐verify level (PV) of the search data, and the resulting *I*_BL_ is monitored. If any stored PV in a string exceeds the applied search PV, that cell operates in the subthreshold regime, suppressing the current of the entire string. Such a case is defined as an *OFF* string, representing a mismatch. Conversely, if all stored PVs are equal to or lower than the search PV, current flows through the string. Within this condition, two distinct cases emerge: (i) when all PVs exactly match the search PV, a moderate *I*_BL_ is obtained, referred to as the *MATCH* string; and (ii) when one or more PVs are lower than the search PV, the *I*_BL_ increases, producing an *ON* string that reflects partial matches. Note that both *ON* and *OFF* strings correspond to mismatch strings. The ability to distinguish *OFF*, *MATCH*, and *ON* string states forms the basis of the TLCAM's associative operation.

To separate these states reliably, the sensing strategy is highlighted in Figure 3B. The SA connected to each BL operates with a defined time window. During sensing, the BL is first precharged, and *I*_BL_ subsequently generates a time‐dependent voltage change at the sense node through charge integration on the sensing capacitance. The resulting voltage is evaluated through simple discrete‐time sampling or comparison. This sensing operation follows the same fundamental principle as conventional V‐NAND flash SAs, with the key distinction being an increased number of sampling events to resolve matching conditions. Consistent with this approach, prior V‐NAND CAM studies [27, 28] have also employed BL discharge speed, evaluated against one or multiple reference levels, to discriminate the string current. *OFF* strings, which generate very low current, fall outside the sensing window and are effectively rejected. Among the remaining strings, the *MATCH* string appears as the one sensed last within the valid window, while *ON* strings are sensed earlier due to their larger *I*_BL_s.

At the array level, the proposed search procedure is performed along the WL direction, as depicted in Figure 3C. This WL‐oriented approach contrasts with earlier DSL‐based search methods and is naturally aligned with the vertical architecture of V‐NAND flash memory. Each DSL plane is processed sequentially, while all BLs within that plane are evaluated in parallel. This design avoids the density loss and fabrication complexity associated with DSL‐directional search, where large numbers of DSLs would otherwise be required, and it reduces the energy overhead by limiting the use of biases on non‐searched DSL planes.

### 2.3 Bottleneck in the Operation of the Proposed V‐NAND TLCAM

While the proposed WL‐directional search scheme enables density‐preserving CAM functionality in V‐NAND, its operation is not free from challenges. A critical bottleneck arises when distinguishing true *MATCH* strings from certain *OFF* strings under specific biasing conditions.

Figure 4A shows the measured BL current–WL voltage (*I*_BL_–*V*_WL_) characteristics of the commercial V‐NAND flash memory used in this study. All data measured in this work were obtained using an unmodified commercial V‐NAND flash memory with more than 100 layers fabricated by SK hynix. In the read operation of V‐NAND flash memory, a read bias (*V*_Read_) is applied to the WL of the selected cell, while a high‐pass bias (*V*_Pass_) is applied to the unselected WLs (Figure S1, Note S2 [29, 30, 31]). When multiple WLs are simultaneously applied at the read bias *V*_Read_, the effective resistance of the entire string increases, resulting in a systematic shift of the *I*_BL_–*V*_WL_ curve toward higher voltages. This effect is highlighted in Figure 4B, where the comparison between a single read WL and ten simultaneously biased WLs clearly shows the rightward shift. To maintain sufficient *I*_BL_ for the search operation, it is therefore necessary to apply an additional offset voltage (*V*_WL, CAM_) on top of the search PV level.

**FIGURE 4:**
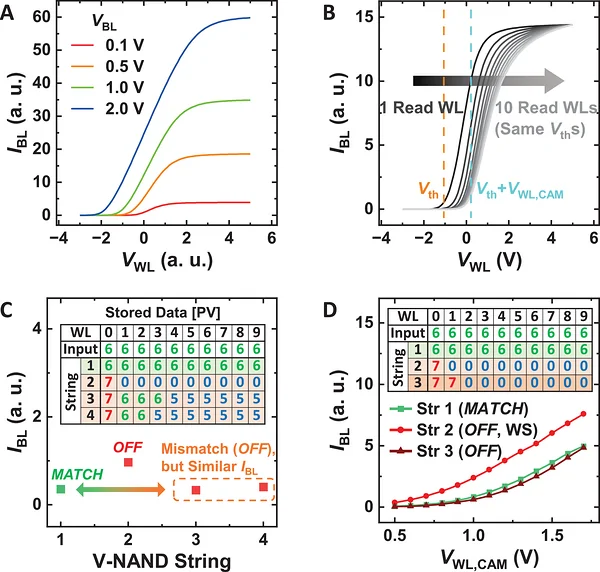
Bottleneck in WL‐directional TLCAM operation. (A) Measured *I*_BL_–*V*_WL_ characteristics of >100‐layer TLC V‐NAND flash memory. (B) Shift of measured *I*_BL_–*V*_WL_ curves when multiple WLs are simultaneously biased, requiring an additional offset voltage (*V*_WL, CAM_). (C) Worst‐scenario (WS) string, where one stored PV slightly exceeds the search PV while others are smaller, producing *I*_BL_ higher than a true *MATCH* string. Inset shows the table of stored PVs and search PV. (D) Dependence of measured *I*_BL_ on stored data with increasing *V*_WL, CAM_, showing that two or more *OFF* cells suppress current below the *MATCH* level.

The presence of this shift introduces a critical worst‐scenario (WS) condition. Figure 4C shows the *I*_BL_ depending on the stored data when the search data is applied simultaneously to 10 WLs. The inset table shows the PVs of the data stored in each WL and the PV of the search data applied as the input. As illustrated in Figure 4C, a WS string is defined as one where only a single stored cell has a PV slightly larger than the search PV, while all other stored cells have significantly smaller PV values. Ideally, such a string should behave as an *OFF* string and produce negligible current. However, due to the overall lower resistance contributed by the majority of low‐PV cells, the string can generate an unexpectedly high *I*_BL_. In practice, as shown in Figure 4C, this WS string current may exceed that of a true *MATCH* string, thereby undermining the reliability of match detection. This overlap stems directly from the cumulative resistance effect observed when multiple WLs are read simultaneously, and it represents a fundamental bottleneck for CAM operation in V‐NAND flash memory.

To address this issue, one intuitive strategy is to increase the number of *OFF* cells within the string. Figure 4D shows the *I*_BL_ depending on the stored data pattern with respect to the additional voltage (*V*_WL, CAM_) applied on top of the search PV level, as defined in Figure 4B. As demonstrated in Figure 4D, when two or more cells store PV values higher than the search PV, the *I*_BL_ is sufficiently suppressed, falling below the level of a *MATCH* string. This observation suggests that redundancy at the cell level can mitigate the WS problem. Building upon this insight, we explored a tied‐WL structure as an alternative.

In the tied‐WL scheme, two adjacent WLs are programmed to store the same data value, thereby ensuring that a mismatch manifests across multiple cells simultaneously. Figure 5A schematically illustrates this structure, where pairs of WLs encode identical PVs. This redundancy ensures that each mismatch is represented by two *OFF* cells, thereby suppressing *I*_BL_ below the *MATCH* level more effectively. Experimental validation of this scheme is presented in Figure 5B,C, which show the *I*_BL_ response as a function of mismatch count and mismatch PV (search PV – stored PV), respectively. The results indicate that the *I*_BL_ increases with both the magnitude of the mismatch PV and the number of mismatched WLs, consistent with the cumulative resistance mechanism. Importantly, the tied‐WL approach ensures that WS strings produce lower currents than *MATCH* strings under all operating conditions.

**FIGURE 5:**
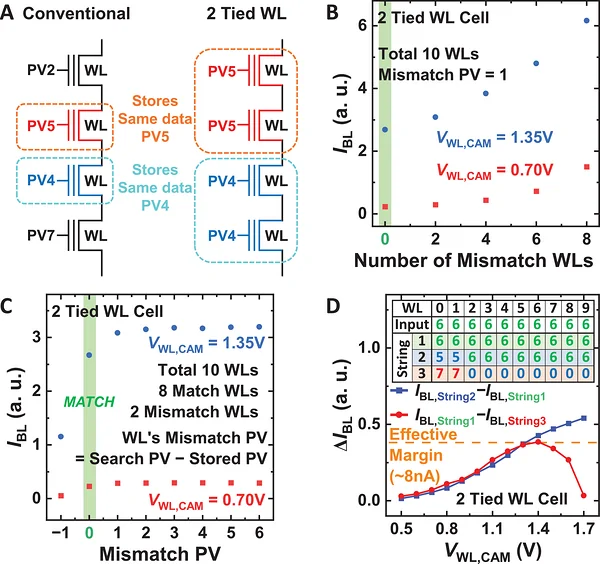
Tied‐WL structure as a redundancy scheme. (A) Schematic of tied‐WL structure where two adjacent WLs store identical data to enforce stronger *OFF* conditions. (B, C) Measured *I*_BL_ response as a function of (B) mismatch WL count and (C) mismatch PV, using a 2‐tied‐WL structure. (D) Comparison of measured sensing margins, showing that *WS*–*MATCH* (red curve) and *ON*–*MATCH* (blue curve) separations remain similar, limiting robustness.

Nevertheless, the tied‐WL structure introduces its own trade‐offs. As summarized in Figure 5D, the margin between WS and *MATCH* strings (red curve) is still limited and remains comparable to the margin between *ON* and *MATCH* strings (blue curve), regardless of *V*_WL, CAM_. For robust sensing, the margin between *OFF* and *MATCH* strings must be significantly larger than the *MATCH*–*ON* separation; otherwise, reliable exclusion of all *OFF* strings through SA tuning becomes impractical (Figure 3B). Furthermore, because each data entry requires two WLs instead of one, the effective storage density of the array is reduced by half, negating one of the primary advantages of the proposed WL‐directional scheme. These observations underscore the necessity of developing alternative approaches that preserve density while providing a sufficiently large sensing margin for robust CAM functionality in V‐NAND flash memory.

### 2.4 Parity‐Split Scheme for Reliable V‐NAND TLCAM Operation

As discussed in the previous section, the most critical bottleneck in the proposed WL‐directional TLCAM arises from the difficulty of reliably distinguishing *MATCH* strings from WS strings. While tied‐WL redundancy mitigates this problem, it does so at the expense of halved storage density. To overcome this trade‐off, we introduce a parity‐split (PS) scheme that enhances discrimination margins without sacrificing array density.

Figure 6A shows the *I*_BL_–*V*_WL_ characteristics under different configurations of simultaneously biased WLs, depending on their physical adjacency. When non‐adjacent WLs are selected together, the effective resistance of the spacer regions is reduced by the pass bias, leading to a lower overall resistance and consequently higher *I*_BL_. Building on this observation, we therefore partition the WLs into odd and even groups. The idea is to reduce string resistance and increase the *I*_BL_ of *MATCH* strings, thereby making them distinguishable from WS strings. However, as demonstrated in Figure 6B, even when only the odd WLs are read, the PV of an *OFF* cell must exceed the search PV by at least two levels to yield a significantly lower *I*_BL_ than that of a *MATCH* string. If the *OFF* PV is only one level higher, the resulting *I*_BL_ may remain comparable to, or even larger than, that of the *MATCH* string, reintroducing ambiguity.

**FIGURE 6:**
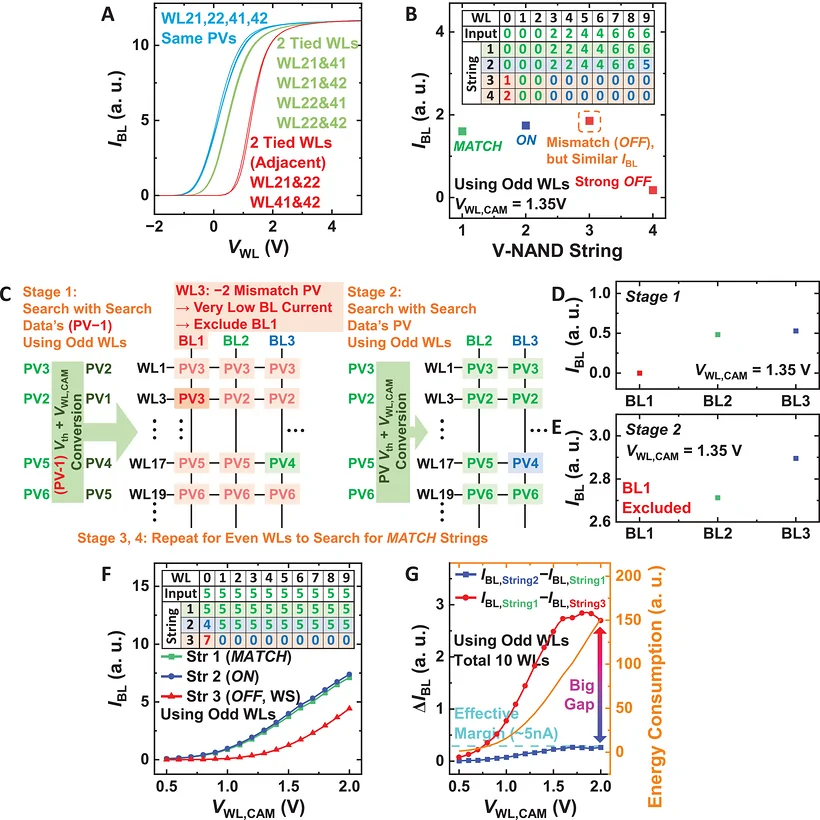
Parity‐split (PS) scheme for reliable TLCAM operation. (A) Measured *I*_BL_–*V*_WL_ characteristics with different WL adjacency configurations; non‐adjacent WLs exhibit reduced resistance. (B) Measured *I*_BL_ characteristics of strings depending on mismatch PV. Even with odd WLs only, *OFF* PV must exceed search PV by ≥2 levels for sufficient suppression. (C) PS scheme operation: staged search using PV–1 then PV for odd WLs, repeated for even WLs. (D, E) Measured *I*_BL_s in (D) Stage 1 and (E) Stage 2 of the PS scheme illustrated in figure 6C. (F) WS strings clearly separated from *MATCH* strings with suppressed currents. (G) Quantified sensing margin and energy consumption as functions of *V*_WL, CAM_: separation between *MATCH* and WS (red curve) exceeds *MATCH*–*ON* (blue curve).

To resolve this, the PS scheme modifies the search sequence, as illustrated in Figure 6C. Instead of directly applying the search PV, the first step is to apply a PV that is one level lower (PV‐1) to the odd WLs. With this lowered threshold, even *OFF* strings with stored PVs just one level above the search PV produce very low current and are effectively excluded from detection. In other words, the margin for excluding *OFF* strings is shifted from a mismatch of –1 level to –2 levels, ensuring that WS strings cannot be mistaken for *MATCH* candidates. After this exclusion step, a second search is carried out with the original PV level, allowing the exact *MATCH* string to be identified among the odd WLs. The same two‐step process is then repeated for the even WLs, resulting in a final reliable *MATCH* identification across the entire block.

Figure 6D,E experimentally verify the PS scheme illustrated in Figure 6C using the same programmed PV distributions and search data. In Stage 1, the search voltages corresponding to one PV lower than the input data are applied to the odd WLs. Owing to the −2 PV mismatch at WL3, the *I*_BL_ of BL1 is almost completely suppressed, whereas BL2 and BL3 maintain relatively high *I*_BL_s, as shown in Figure 6D. Therefore, the string connected to BL1 is identified as a strongly mismatched string and excluded from the subsequent search operation. In Stage 2, the search voltages corresponding to the input PVs are applied to the remaining strings. As shown in Figure 6E, BL2 and BL3 exhibit high *I*_BL_s, while BL1 is not evaluated because it was already excluded in Stage 1. These measured results validate the sequential search scheme, in which strongly mismatched strings are eliminated at an early stage, and only the remaining candidate strings proceed to the subsequent search stages. In Stage 2, BL2 corresponds to a *MATCH* current, whereas BL3 exhibits an *ON* current; therefore, the *I*_BL_ of BL3 is slightly higher than that of BL2.

The effectiveness of the PS scheme is confirmed in Figure 6F,G. As shown in Figure 6F, the WS string can be clearly separated from the *MATCH* string, with *OFF* currents suppressed below the detection threshold. Moreover, Figure 6G quantifies the sensing margin: the separation between *MATCH* and WS strings (red curve) is significantly larger than the separation between *MATCH* and *ON* strings (blue curve). This enhanced margin enables the SA to robustly filter out *OFF* strings without compromising detection of true matches. The *ON* strings, characterized by slightly larger *I*_BL_s, can still be discriminated based on their shorter sensing times.

An important design trade‐off arises from the choice of additional WL bias (*V*_WL, CAM_). Increasing *V*_WL, CAM_ enlarges the sensing margin and improves robustness, but it also leads to higher energy consumption. In this work, two representative bias conditions were evaluated: a lower voltage of 0.70 V and a higher voltage of 1.35 V. As shown in Figure 6G, 1.35 V provides a sufficient margin (on the order of several nA) for reliable discrimination [27, 28], whereas 0.70 V offers reduced energy overhead but smaller separation between string states. Because the *I*_BL_–*V*_WL_ characteristics of different PV states exhibit approximately parallel voltage shifts, a common *V*_WL, CAM_ can be used across the TLC levels without PV‐specific calibration.

### 2.5 Operation of V‐NAND TLCAM With the PS Scheme

The proposed PS scheme was experimentally verified to confirm its ability to provide robust and flexible CAM functionality in V‐NAND flash memory. Figure 7 summarizes the operation of the TLCAM using the PS approach under different mismatch and “don't‐care” conditions.

**FIGURE 7:**
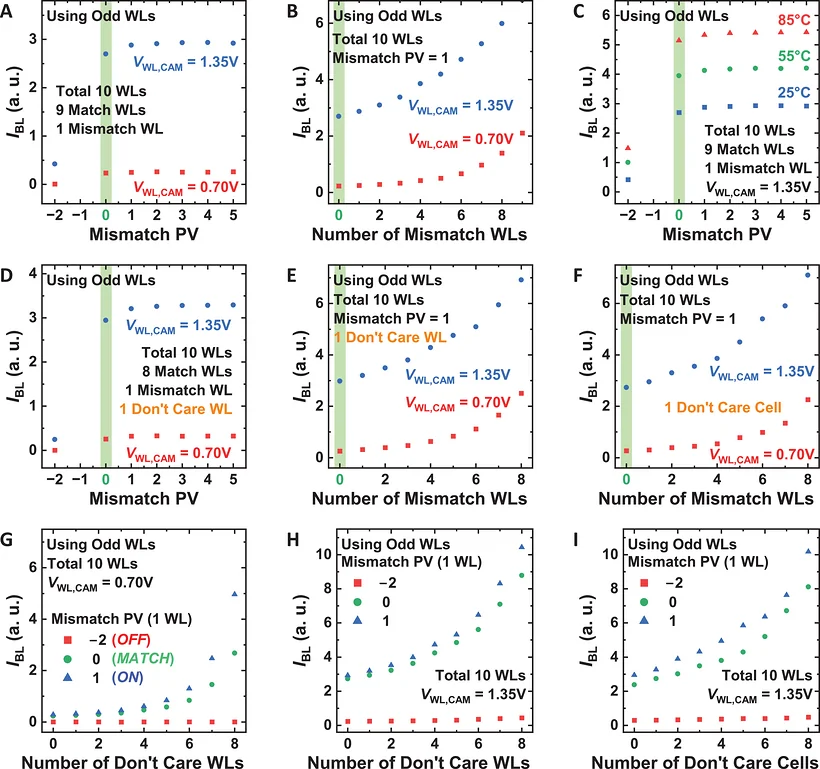
Operation of TLCAM with PS scheme under mismatch and don't‐care conditions. (A, B) Measured *I*_BL_ with odd WLs selected, showing stable separation regardless of (A) mismatch PV and (B) number of mismatch WLs. (C) Temperature dependence of *I*_BL_ as a function of the mismatch PV. (D, E) CAM response with designated “don't‐care” WLs excluded by pass bias and (F) “don't‐care” cells set to a fully erased state, preserving reliable *MATCH*‐string detection. (G, H) Effect of increasing number of “don't‐care” WLs and (I) “don't‐care” cells: reduced string resistance slightly raises *I*_BL_, but *OFF*–*MATCH* margin remains sufficient for robust discrimination, regardless of *V*_WL, CAM_. The don't‐care cells are biased with the lowest PV (PV0).

Figure 7A,B show the measured *I*_BL_ of the TLCAM under the PS scheme for various mismatch conditions. In accordance with the PS scheme, only the odd WLs were selected. The displayed range begins at −2 because this is the closest and most critical *OFF* condition produced during the PV‐staged search, whereas larger negative or positive mismatches yield more strongly suppressed or enhanced currents and are therefore more readily distinguishable from the *MATCH* state. An *OFF* string with a negative mismatch PV exhibits a significantly lower *I*_BL_ compared to a *MATCH* string with a mismatch PV of 0. *MATCH* strings consistently exhibit moderate current levels, while *ON* strings (where stored PVs are lower than the search PV) generate larger *I*_BL_s. These results validate that the PS scheme maintains stable separation between *OFF*, *MATCH*, and *ON* strings even as mismatch complexity increases, thereby preserving accurate associative search behavior. The temperature dependence of the CAM search operation was further evaluated over a temperature range of 25°C–85°C as shown in Figure 7C. As the temperature increased, *I*_BL_ increased over the entire mismatch‐PV range owing to the enhanced channel conduction at elevated temperatures. Nevertheless, the characteristic current separation was consistently maintained at all measured temperatures.

Beyond mismatch handling, the PS scheme also supports the integration of “don't‐care” bits. For search wildcards, selected WLs can be effectively excluded from comparison by applying a pass bias. Figure 7D,E illustrate the TLCAM response when one or more WLs are designated as “don't‐care.” In these cases, the corresponding WLs no longer contribute to the match decision, providing flexibility for wildcard operations in search queries. The results confirm that the PS scheme continues to identify *MATCH* strings reliably, even when part of the search word is masked by “don't‐care” states. In addition to input wildcards, the proposed TLCAM also supports stored wildcards at the cell level. A “don't‐care” cell is realized by adjusting the cell into a fully erased state below PV0, i.e., by leaving the cell unprogrammed after the block erase operation without any subsequent program step. In this state, the cell exhibits saturation current for all applied search PV levels and therefore matches any input value. Figure 7F demonstrates that the stored wildcard operates correctly, in the same manner as the search wildcard. In these measurements, the WLs containing the don't‐care cells were driven with the lowest search PV (PV0).

An important consideration is the electrical impact of assigning multiple “don't‐care” WLs or cells. As shown in Figure 7G,H, increasing the number of WLs under pass bias reduces the overall resistance of the V‐NAND string, leading to a modest increase in *I*_BL_. However, the measured data indicate that this current increase is minimal for *OFF* strings and does not compromise the ability to distinguish them from *MATCH* strings. The effective sensing margin between *OFF* and *MATCH* conditions remains sufficiently large to ensure robust match detection. Moreover, the *ON* string behavior, although influenced by reduced resistance, can still be separated by its faster response time relative to *MATCH* states. Figure 7I presents the corresponding results for stored wildcards (don't‐care cells) and exhibits the same trend as observed in Figure 7H, confirming that the electrical impact and sensing robustness are consistent for both wildcard implementations.

### 2.6 Reliability and Efficiency of the V‐NAND TLCAM

To evaluate the reliability of the proposed TLCAM under diverse search conditions, including the impact of *V*_th_ variation, additional measurements were performed using randomly generated search data. Random combinations of stored data and input queries were generated, and the corresponding *I*_BL_s were measured. To faithfully reflect practical V‐NAND operation, all data are programmed using the standard incremental‐step‐pulse‐programming (ISPP) scheme employed in commercial devices [23, 32], with a program‐voltage step size of 0.5 V. Because ISPP repeatedly applies program and verify operations until each cell reaches its designated *V*_th_ window, cell‐to‐cell variations, program efficiency, and cycling‐dependent programming characteristics primarily alter the required number of program pulses. Once the target *V*_th_ is established, the TLCAM search response is governed by the achieved *V*_th_ relative to the applied search voltages, rather than by the programming history. Thus, the evaluated distributions include the residual *V*_th_ dispersion after ISPP verification.

Figure 8A shows the separation of *I*_BL_s among *OFF* (WS), *MATCH*, and *ON* strings under ISPP‐induced *V*_th_ variation and randomly generated search conditions. The *ON* string contains a single cell with a ‘1’ mismatch, while the *OFF* string is configured as the WS condition; together, these represent the cases that yield *I*_BL_ values closest to that of a *MATCH* string. Despite the presence of *V*_th_ dispersion, the *ON* strings consistently exhibit higher *I*_BL_s than the *MATCH* strings, with an average current separation of approximately 4.5 nA (consistent with the effective margin observed in Figure 6G), while the WS *OFF* strings remain clearly distinguishable at substantially lower current levels. This analysis is further extended to configurations with different numbers of “don't‐care” WLs. For each configuration, random search data are generated and programmed using the same 0.5 V‐step ISPP procedure. As shown in Figure 8B, reliable discrimination among *OFF*, *MATCH*, and *ON* strings is maintained regardless of the number of masked WLs. Across 100 randomly generated search‐data patterns, the *I*_BL_ of the *ON* string was consistently higher than that of the *MATCH* string in every case. These results confirm that the proposed TLCAM operation remains reliable across a wide range of search conditions and stored data patterns.

**FIGURE 8:**
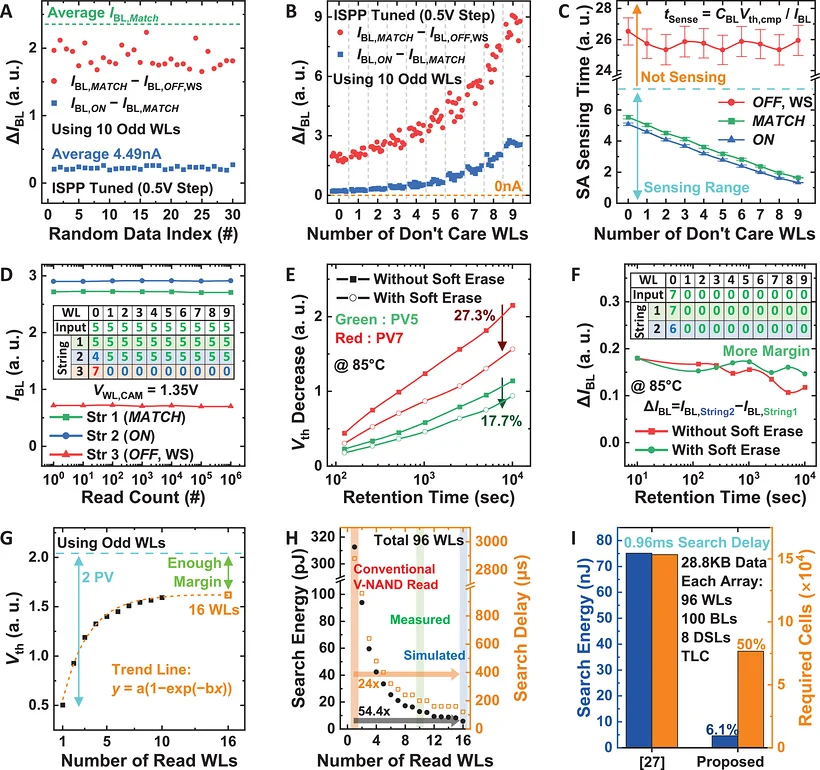
Energy efficiency and reliability of the proposed TLCAM. (A) *I*_BL_ differences between *MATCH* and *OFF*(WS)/*ON* string under randomly generated search data. The *ON* string contains a single cell with a mismatch of 1. (B) *I*_BL_ difference characteristics as a function of the number of don't‐care WLs under random search data. (C) Calculated SA sensing times for *MATCH*, *ON*, and *OFF* strings. The search data were randomly generated, and the error bars represent the standard error. (D) *I*_BL_ stability measured across repeated read cycles, confirming robust operation. (E, F) Measured retention characteristics: (E) *V*_th_ drift and (F) sensing margin reduction over time, most pronounced at PV7. Applying a soft erase after programming mitigates charge loss, maintaining higher margins over extended retention. (G) Increase in *V*_th_ with number of simultaneously read WLs, showing reduced margin for WS discrimination. Trend extrapolation indicates feasibility up to 16 WLs. (H) Search energy and delay comparison: conventional single‐WL readout, measured 10‐WL, and simulated 16‐WL cases. Proposed TLCAM achieves 54.4× energy savings and 24× speedup. (I) Comparison of previous work [27] and the proposed TLCAM. Proposed TLCAM achieves 93.9% reduction in search energy and 50% area consumption.

In addition to current‐domain analysis, the corresponding SA sensing‐time characteristics are derived based on the measured *I*_BL_ values under randomly generated search conditions, as shown in Figure 8C. The *MATCH* strings are consistently detected more slowly than the *ON* strings, while the *OFF* strings are detected significantly later, exhibiting a large temporal separation. Moreover, the current difference between the *ON* and *MATCH* strings corresponds to approximately 10% of the *MATCH* string current (Figure 8A), providing a sufficiently large margin to enable robust and reliable discrimination using the SA in the time domain.

The robustness of the proposed scheme under repeated operations was also examined. Figure 8D shows the stability of the *I*_BL_ across repeated read cycles. Even as the read count increases, the TLCAM maintains consistent separation between *OFF*, *MATCH*, and *ON* string conditions, confirming stable operation under repeated use.

Reliability over extended retention times was further evaluated. The PS scheme modifies only the read‐bias sequence by separately accessing odd and even WLs and does not require additional program or erase operations; therefore, it does not intrinsically alter the retention characteristics of the stored data. A known challenge in V‐NAND flash memory is that cells programmed to higher PV states (corresponding to higher *V*_th_ values) exhibit larger *V*_th_ degradation during retention [33, 34], leading to a gradual reduction in sensing margin. Figure 8E,F illustrate the evolution of *V*_th_ and the corresponding TLCAM sensing margin as a function of retention time. The measurements included cells programmed to the highest state (PV7), which shows the largest *V*_th_ reduction. Over time, charge loss induces a gradual decrease in *V*_th_, which in turn reduces the sensing margin of the TLCAM. To alleviate this degradation, a soft‐erase step was applied after programming. This process removes shallowly trapped electrons, thereby suppressing the rate of charge loss [35]. As a result, the sensing margin is better preserved across long retention times. While the margin remains positive even without soft erase, ensuring reliable CAM operation, the application of soft erase enables the margin to be maintained at a consistently higher level, providing improved robustness against retention‐induced degradation.

One of the key advantages of implementing content‐addressable functionality directly within V‐NAND is the potential to significantly reduce both search latency and energy consumption. Figure 8G shows the measured increase in *V*_th_ as a function of the number of WLs involved in a single search operation. As the number of simultaneously read WLs increases, the effective resistance of the V‐NAND string also increases. This resistance rise reduces the sensing margin available to distinguish WS strings from true *MATCH* strings. In our experiments, measurements were conducted using up to 10 odd WLs, limited by the available contact pads. Nevertheless, extrapolation of the trend line indicates that even with a conservative design approach, simultaneous search across 16 odd WLs is feasible without margin collapse. Accordingly, within the conservatively selected 16‐WL search group, increasing the number of don't‐care WLs does not become the limiting factor because the *OFF*, *MATCH*, and *ON* strings remain distinguishable; instead, the primary constraint is the total number of WLs simultaneously activated. By employing the PS scheme, this enables 32 WLs in total (16 odd and 16 even) to be searched in just four read operations. Note that a TLC V‐NAND flash cell (3‐bit) requires three read operations to determine which of the eight states it resides in. Compared to conventional single‐WL readout, this corresponds to a 24× improvement in search throughput.

Beyond throughput, the simultaneous activation of multiple WLs provides substantial energy savings. Figure 8H compares the search energy and delay for three representative cases: conventional single‐WL readout (read operation of V‐NAND flash memory), measured 10‐WL parallel read, and simulated 16‐WL parallel read. The results confirm that a 16‐WL parallel read using the proposed TLCAM achieves a 54.4× reduction in energy consumption together with a 24× reduction in search delay relative to the conventional baseline (Note S3 [36, 37]). Note that the energy values account only for current conduction through the selected V‐NAND strings under the applied read biases. They do not include the energy consumed by the SAs and their repeated sampling events, WL drivers, reference‐level generation, data encoding or decoding, or peripheral control logic. Therefore, in the case of the TLCAM, the increased number of SA sampling events may result in a slight increase in energy consumption. These improvements originate from both reduced per‐search cycle overhead and enhanced parallelism across the WL stack. The reduction in *I*_BL_ caused by reading multiple WLs simultaneously also contributed to the improvement in energy consumption.

In addition, a quantitative comparison with previously reported V‐NAND CAM architectures [27] is performed in terms of search latency, energy consumption, and array area, as summarized in Figure 8I. A representative TLC V‐NAND array consisting of 96 WLs, 100 BLs, and 8 DSLs is considered, corresponding to a storage capacity of 28.8 KB per array when fully populated [38, 39]. In this comparison, the proposed TLCAM performs each search by simultaneously activating 16 WLs, whereas prior V‐NAND CAM architectures encode a single data bit using two adjacent WL cells and execute search operations by activating all DSL planes simultaneously. Under these assumptions, both architectures achieve comparable search latency by design, as the effective number of read cycles is matched in the comparison.

Under these matched‐latency conditions, a pronounced difference is observed in search energy consumption. In prior V‐NAND CAM architectures, all DSL planes are activated during each search operation, causing all strings within the block to contribute to the total energy consumption at every read cycle. In contrast, the proposed TLCAM activates only a single DSL plane per search, while the remaining DSL‐connected strings remain unselected and contribute only negligible leakage currents (∼pA). As a result, when normalized to the same search latency, the proposed TLCAM consumes only approximately 6.1% of the search energy required by prior V‐NAND CAM architectures. Beyond energy efficiency, the proposed TLCAM also provides a clear advantage in array area efficiency. Prior V‐NAND CAM architectures inherently require two flash cells to store a single data bit, leading to a 2× increase in array area for the same storage capacity. In contrast, the proposed TLCAM stores one 3‐bit data unit per cell without redundancy, thereby preserving the intrinsic density advantage of the V‐NAND array. The results strongly support the potential of TLCAM to deliver large‐scale, energy‐efficient associative search directly within commercial V‐NAND flash arrays.

### 2.7 Few‐Shot Learning With the Proposed V‐NAND TLCAM

To further evaluate the system‐level potential of the proposed V‐NAND TLCAM, we applied it to a few‐shot learning (FSL) task using the Omniglot dataset, which is widely used as a benchmark for FSL [5, 11]. In FSL, the goal is to classify new categories with only a handful of labeled examples. For an *N*‐way *K*‐shot task, the system is given *K* examples from each of *N* classes (the support set), and must then identify the class of a new query image by comparing it with the stored support samples. This paradigm mimics the ability of biological learning systems to generalize from very few examples and has been considered an important step toward building intelligent, adaptive machines.

Figure 9A,B show the transient characteristics of the TLCAM's *I*_BL_ under different mismatch conditions. These measurements correspond to the *I*_BL_ characteristics depending on mismatch conditions shown earlier (Figure 7A,B), but are now observed in the time domain. These transient responses were incorporated into the FSL simulation framework by modeling the impact of read noise on match detection.

**FIGURE 9:**
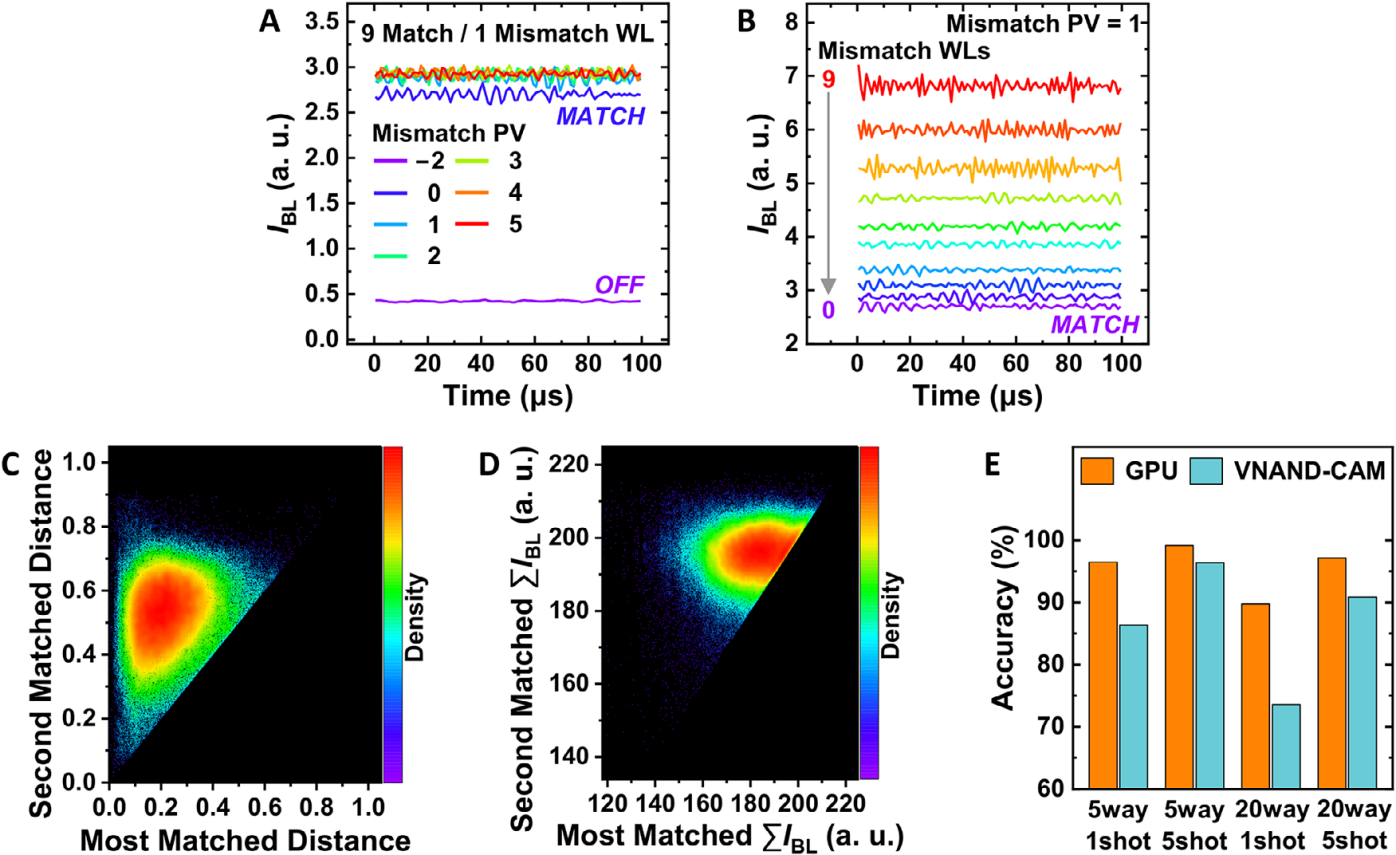
Few‐shot learning demonstration with V‐NAND TLCAM. (A, B) Transient *I*_BL_ waveforms under different (A) mismatch PVs and (B) mismatch WL counts, corresponding to steady‐state characteristics in figure 7A,B. (C) Scatter plot of most vs. second matched distances in 20‐way 5‐shot learning using GPU+cosine. (D) Scatter plot using TLCAM+LSH; distances represented by aggregate BL current. (E) Classification accuracy on Omniglot for GPU+cosine and TLCAM+LSH across 5‐way/20‐way and 1‐shot/5‐shot tasks. TLCAM shows reduced accuracy in 1‐shot, but achieves >90% in 5‐shot, comparable to the GPU baseline.

The FSL simulation setup follows the methodology in prior studies [5, 11], but is adapted to the TLCAM architecture (Note S4 [5, 11]). For the GPU baseline, features were first extracted from Omniglot images using a convolutional neural network (CNN). The resulting real‐valued feature vectors were then compared directly on the GPU by computing the cosine similarity between each query and the support set. For the proposed TLCAM approach, the same CNN feature embeddings were projected by a Gaussian random matrix and subsequently quantized into 8 TLC levels (0–7). The quantized PV signatures were organized into 16‐WL parity‐split groups. We used locality‐sensitive hashing (LSH) for the TLCAM approach. For each group, the measured current–voltage characteristics (stored as a lookup table indexed by mismatch PV and mismatch count) were used to assign the *I*_BL_, including experimentally observed noise modeled with Gaussian perturbations. The group *I*_BL_ values were then summed to produce an aggregate *I*_BL_, which served as the similarity score: a smaller total *I*_BL_ corresponds to a higher similarity between the query and support sample. During inference, each query vector was compared against all stored support signatures in the TLCAM, and the nearest neighbor was identified as the support entry with the minimum aggregate *I*_BL_.

Figure 9C,D present scatter plots of the most matched distance vs. the second matched distance in a 20‐way 5‐shot learning task, intended to qualitatively illustrate the decision margin between the top‐ranked and second‐ranked candidates within each respective similarity metric. For the GPU+cosine baseline (Figure 9C), data points lie exclusively above the *y = x* line, as expected, reflecting that the closest match always has a smaller distance than the second closest. In the TLCAM+LSH case (Figure 9D), the distances are represented by the sum of *I*_BL_s rather than cosine metrics. While most data remain above the *y = x* line, more points cluster near the diagonal, indicating that the most matched and second matched candidates are often less separable. This suggests that TLCAM‐based FSL may suffer from degraded accuracy in certain cases due to smaller decision margins.

Figure 9E compares the classification accuracy across different way‐ and shot‐settings for GPU‐ and TLCAM‐based implementations. As expected, in the 1‐shot regime, TLCAM exhibits lower accuracy than the GPU baseline because of reduced separation between nearest candidates. The decline in accuracy is likely influenced by the PS scheme's strategy of completely excluding *OFF* strings. While this approach is effective for identifying stored data that exactly matches the search data, it can be disadvantageous in FSL, where the goal is to retrieve similar rather than identical data. However, in the 5‐shot regime, the performance gap narrows significantly, with TLCAM achieving over 90% accuracy (90.85%, 20‐way 5‐shot) and showing results comparable to the GPU implementation. This reflects the fact that multiple support examples per class improve robustness against device‐level noise and variations. Taken together, these results confirm that the proposed V‐NAND TLCAM is capable of supporting FSL with competitive accuracy, especially in multi‐shot settings.

### 2.8 Benchmark Comparison With Prior CAM Technologies

Table 1 benchmarks the proposed V‐NAND TLCAM against representative CAM implementations based on V‐NAND flash memory [27, 40], FeFETs [5, 41], and memristors [3]. Previous V‐NAND‐based approaches achieve multi‐level or analog‐range search by encoding one comparison element across two flash cells. In the complementary‐storage scheme [40], the target value and its complementary value are stored in two adjacent NAND cells. Likewise, the analog in‐memory‐search architecture [27] uses two flash cells to define the lower and upper boundaries of a valid input range. Although these approaches enable multi‐level search, their two‐cell encoding increases the number of physical cells required for each comparison element, thereby reducing the effective storage density relative to the native NAND array. In addition, query‐side and stored‐data don't‐care operations were not explicitly demonstrated in these implementations.

**TABLE 1:** Comparison of the proposed V‐NAND TLCAM with prior CAM implementations.

	This work	Wang [40]	Tseng [27]	Ni [5]	Yin [41]	Li [3]
Device	Vertical NAND	Vertical NAND	Vertical NAND	FeFET	FeFET	TaO_x_ memristor
Multi‐level storage	Yes	Yes	Yes	No	Yes	Yes
CAM cell configuration	1 cell	2 cells	2 cells	2 FeFETs	2 FeFETs	6 Transistors 2 Memristors
Query‐side don't‐care support	Yes	No	No	Not reported	Not reported	Not reported
Stored‐data don't‐care support	Yes	No	No	Yes	Yes	Yes

Emerging‐device CAMs exhibit different trade‐offs between functionality and cell complexity. The FeFET TCAM [5] implements a stored don't‐care state using two FeFETs, but supports binary or ternary rather than multi‐level data storage. In contrast, the FeFET‐based analog CAM [41] stores analog decision boundaries using two FeFETs and supports a full‐range stored state that functions as a stored‐data don't‐care condition. The memristor‐based analog CAM [3] also enables multi‐level range matching and stored‐data don't‐care operation, but requires a 6‐transistor–2‐memristor cell. Query‐side don't‐care functionality was not explicitly reported in these implementations.

In comparison, the proposed TLCAM directly exploits the eight intrinsic *V*_th_ states of a single TLC V‐NAND flash cell, thereby enabling multi‐level associative search without an additional complementary storage cell. Query‐side don't‐care operation is implemented by applying a pass bias to the corresponding WL, whereas stored‐data don't‐care operation is realized using a fully erased cell state. Consequently, among the compared implementations, the proposed V‐NAND TLCAM uniquely combines single‐cell multi‐level storage with both query‐side and stored‐data don't‐care functionality. This combination preserves the native storage‐density advantage of commercial V‐NAND flash memory while providing flexible associative‐search capability.

## 3 Discussion

The results presented in this work demonstrate that commercial V‐NAND flash memory, originally developed for high‐density data storage, can be repurposed as an efficient platform for associative computing. By leveraging the triple‐level states of TLC cells, the proposed V‐NAND TLCAM achieves compact search functionality with enhanced flexibility compared to binary and ternary CAMs. The introduction of the PS scheme addresses one of the most critical challenges in multi‐WL operation–‐the WS condition where a partially mismatched string produces higher current than a true match. By separating odd and even WLs and employing a PV‐staged search, the PS scheme provides sufficient sensing margins while preserving the storage density of the array.

Experimental verification further confirmed that the TLCAM maintains stable discrimination of *OFF*, *MATCH*, and *ON* states under various mismatch and don't‐care conditions, highlighting the robustness of the architecture. Moreover, multi‐WL activation demonstrated not only throughput gains but also substantial energy savings, with up to 54× reduction compared to conventional sequential read operation of V‐NAND flash memory. The proposed TLCAM also maintained a sufficient sensing margin for reliable operation even under repeated reads and extended retention times. These results underscore the unique advantage of aligning CAM search operations with the vertical WL architecture of V‐NAND.

At the system level, the evaluation of FSL tasks highlights the broader applicability of the proposed architecture. The TLCAM exhibited reduced margins between nearest candidates in 1‐shot learning, leading to lower accuracy than GPU‐based cosine similarity. However, in 5‐shot tasks, the performance gap narrowed significantly, and the TLCAM maintained over 90% accuracy on the Omniglot dataset. The ability to support competitive accuracy in multi‐shot settings demonstrates that V‐NAND TLCAM can serve as a practical accelerator for similarity‐driven machine learning tasks.

Looking forward, several opportunities remain for further optimization. First, integrating larger‐scale TLCAM arrays with optimized peripheral sensing circuitry could extend the demonstrated benefits to system‐level accelerators for search‐intensive workloads. Second, hardware–software co‐design incorporating optimized LSH projections, nonuniform quantization boundaries, and PV mapping based on the measured *I*_BL_ characteristics could improve decision margins, particularly in 1‐shot learning. Joint optimization with SA sampling thresholds and reference timing may further enhance the separation between the closest candidates and narrow the accuracy gap relative to the GPU baseline. Finally, exploring hybrid architectures that integrate TLCAM with other in‐memory computing primitives may further expand the role of V‐NAND flash memory in future AI hardware.

## 4 Methods

### 4.1 V‐NAND Flash Memory for Experiments

The devices employed in this study are unmodified commercial TLC V‐NAND flash memory provided by SK hynix, consisting of more than 100 WL layers and a total storage capacity of 512 GB (Note S1 [24, 25, 26]). No physical modifications were made to the memory devices or peripheral circuitry, including the cell structure, WL stack, drain/source select devices, or SAs.

### 4.2 Electrical Characterization

Device characteristics were evaluated using a semiconductor parameter analyzer (Keysight B1500A) integrated with a probe station. The analyzer is linked to a switching matrix (B2201A), which routes the applied voltages to the designated contact pads. This configuration enables array‐level measurements with simultaneous multi‐WL driving and controlled BL, DSL, SSL, and SL biasing.

### 4.3 V‐NAND Flash Memory Operations

Standard program, read, and GIDL erase operations were performed with a conventional pulse protocol (Note S2 [29, 30, 31]).

### 4.4 Energy Evaluation

All energy values were calculated for the V‐NAND array read phase only (Note S3 [36, 37]). The array‐core read energy was estimated from the measured *I*_BL_ under the applied read biases and the corresponding read duration and was summed over the active strings and the required read steps. The same energy definition was applied to all compared cases.

## Author Contributions

S.H.P. proposed the idea, performed the experiments, and wrote the paper. M.O., R.H.K., J.I., and D.S. designed the experiments. M.O., Y.Y., and G.J. set up the experimental protocol. J.K. performed the few‐shot learning simulation. J.H.L. supervised the project. All the authors discussed the results and commented on the manuscript.

## Code Availability Statement

The code used for the FSL simulation is available from the corresponding author upon reasonable request.

## Conflicts of Interest

The authors declare no conflicts of interest.

## Supporting information




**Supporting File**: advs77390‐sup‐0001‐SuppMat.pdf.

## Data Availability

The data that support the findings of this study are available from the corresponding author upon reasonable request and with permission of SK hynix Inc.
