# Supplementary material for: Triple‐Level Content‐Addressable Vertical NAND Flash Memory
Source: Adv Sci (Weinh). 2026 Aug 24;13(61):e77390. doi: 10.1002/advs.77390 (PMC13502362; doi:10.1002/advs.77390)
Supplement: Supplementary file 1 [file ADVS-13-e77390-s001.pdf]

# Supplementary Information

**Triple-Level Content-Addressable Vertical NAND Flash Memory**

Sung-Ho Park[1], Jiseong Im[1], Yeongheon Yang[2], Ryun-Han Koo[1], Jonghyun Ko[1], Mingyun Oh[1], Dongbeen Shin[1], Gyuweon Jung[1], and Jong-Ho Lee[1*]

[1]*Department of Electrical and Computer Engineering and Inter-university Semiconductor Research Center, Seoul National University, Seoul 08826, Republic of Korea*
[2]*Research and Development Division, SK hynix Inc., Icheon 17336, Republic of Korea*

[*] Corresponding author. Tel.: +82-2-880-1727; Fax: +82-2-882-4658
E-mail address: jhl@snu.ac.kr (J.-H. Lee)

## Supplementary Note S1

**Architecture of V-NAND flash memory.** In this study, we utilize commercial TLC V-NAND flash memory devices manufactured by SK hynix. The architecture is based on vertically stacked word-lines (WLs), with the device employed here incorporating more than 100 layers. As illustrated in Fig. 2A, a V-NAND string is defined by the configuration of the bit-line (BL) and the drain-select-line (DSL). The source-select-line (SSL) and source-line (SL) are symmetrically placed with respect to the WL stack alongside the DSL and BL, respectively.

Each V-NAND string comprises a polycrystalline Si channel, tunneling oxide, charge-trap nitride, and blocking oxide, where data is stored through electron trapping in the charge-trap nitride (Fig. 2B). The vertically stacked WLs share both the channel and charge-trap nitride within the same string. A V-NAND flash memory cell is defined by combining the WL with the string specified by the BL and DSL, thereby forming the basic unit for information storage in V-NAND flash memory.

The V-NAND flash memory used in this work is an unmodified, off-the-shelf commercial TLC V-NAND flash memory with more than 100 layers and a total storage capacity of 512 GB. The device is identical to the V-NAND flash memory used in our group's previous studies[24–26].

**Supplementary Note S2**

**V-NAND flash memory operations.** The fundamental operations of V-NAND flash memory consist of program, read, and erase processes, as shown in Supplementary Fig. S1. As programming and reading are performed at the level of individual memory cells, proper inhibition of unselected cells is essential during these operations.

The read operation is performed by biasing the selected BL and DSL to allow current conduction through the designated string. A read voltage ($V_{Read}$) is applied to the selected WL, while the unselected WLs are biased with a high pass voltage ($V_{Pass\_R}$) to ensure they remain fully conductive. The BL current corresponding to the selected WL state is then measured.

For programming, a positive bias is applied to the selected WL, inducing electron injection from the channel into the charge-trap nitride. This is achieved by applying a program voltage ($V_{PGM}$) to the target WL; a larger $V_{PGM}$ enhances the potential difference between the channel and the WL, thereby strengthening the programming effect. To suppress programming in the unselected WLs, a pass voltage ($V_{Pass\_P}$) is applied, which transfers the channel potential to the selected WL while minimizing the channel–WL potential difference of unselected cells. On the BL side, the selected BL is biased at 0 V to maximize channel–WL potential difference, whereas a pass bias ($V_{uBL\_P}$) is provided to unselected BLs to prevent unwanted programming even under $V_{PGM}$.

The erase function is implemented using a gate-induced drain leakage (GIDL) mechanism, which applies a positive voltage ($V_{ERS}$) to the BL (channel) in contrast to the program scheme[29-31]. The voltage difference between DSL and BL generates GIDL, producing holes that drift from the channel toward the WL and recombine with stored electrons, thus erasing the trapped charge. Erase is executed at the block level, requiring all WL, BL, and DSL lines to be active. Specifically, voltages are applied to BL and DSL to produce the required holes, with the voltage gap ($V_{GIDL} = V_{ERS} - V_{DSL\_E}$) governing the hole generation rate. Increasing $V_{GIDL}$ strengthens

the erase process. Identical voltages are also applied to the SL and SSL to induce GIDL, while the WLs are kept at 0 V to enable hole transport driven by the channel–WL potential difference. As $V_{ERS}$ increases, the channel potential rises, thereby enhancing the erase efficiency.

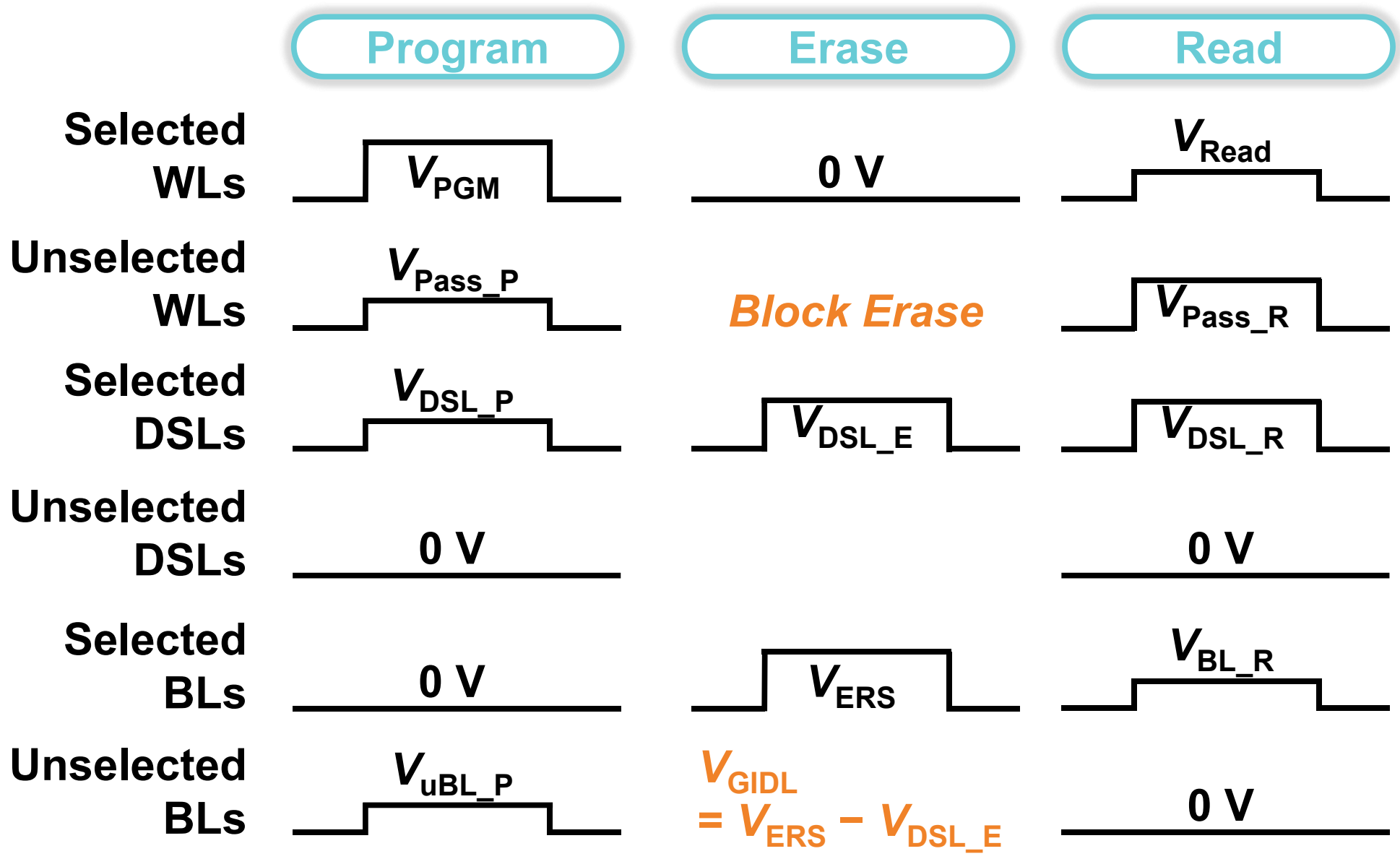


**Supplementary Fig. S1** Pulse schemes of V-NAND flash memory.

**Supplementary Note S3**

**Calculation of delay and energy consumption in V-NAND TLCAM.** To evaluate the improvements in search delay and energy consumption achieved by the proposed V-NAND TLCAM and the parity-split (PS) scheme, the following equations were used[36,37]:

$$t_{\mathrm{Delay}} = t_{\mathrm{Read}} \times n_{\mathrm{Read}}$$

$$E_{\mathrm{String}} = V_{\mathrm{BL}} \times I_{\mathrm{BL}} \times t_{\mathrm{Read}} \times n_{\mathrm{Read}}$$

Here, $t_{\mathrm{Delay}}$ denotes the total search delay, and $E_{\mathrm{String}}$ represents the energy consumed within a single V-NAND string. $t_{\mathrm{Read}}$ is the time required for a single read operation, and $n_{\mathrm{Read}}$ is the number of read cycles required to search across 96 WLs. The calculated results correspond to the delay and energy consumption necessary for searching a single V-NAND string consisting of 96 WLs, as presented in Fig. 8H.

The energy consumption of the prior V-NAND CAM[27] is also calculated using the same methodology and assuming the same read time ($t_{\mathrm{Read}}$) as that of the proposed TLCAM.

## Supplementary Note S4

**FSL simulation methodology for V-NAND TLCAM.** We evaluated the system-level behavior of the proposed V-NAND TLCAM on few-shot learning (FSL) tasks using the Omniglot dataset, following the episodic evaluation protocol widely adopted in prior memory-augmented neural network (MANN) studies[5,11]. The Omniglot dataset contains 1,623 handwritten characters from 50 different alphabets, with 1,200 characters used for training and the remaining characters reserved for testing. Each class contains only 20 examples, making the dataset a challenging benchmark for few-shot learning because of its large number of categories and small per-class sample size. In an $N$-way, $K$-shot task, the system is provided with a support set consisting of $K$ examples from each of $N$ classes and is then required to classify a novel query image from among those $N$ classes.

The FSL simulation setup in our work follows the methodology established in prior CAM-based approaches but is adapted to the TLCAM architecture. For the GPU baseline, features were extracted from Omniglot images using a convolutional neural network (CNN), and the resulting real-valued embeddings were compared directly on the GPU by computing cosine similarity between each query and the support set. For the TLCAM-based approach, we introduced an additional locality-sensitive hashing (LSH) layer, which replaces the final fully connected layer of the CNN. Instead of feeding features into a softmax classifier, the LSH layer performs a random projection of the CNN embeddings followed by quantization. In conventional TCAM-based MANNs, this procedure converts real-valued features into compact binary signatures; in our TLCAM design, the projected values were quantized into eight program-verify (PV) levels (0–7), thereby mapping features to signatures directly compatible with the TLC flash device.

**Supplementary Note S5**

**Quantization-resolution sensitivity of LSH-based few-shot learning.** To quantify the tolerance of the LSH-based few-shot learning framework to quantization resolution, we additionally evaluated the classification accuracy by varying the number of quantization levels applied to the projected CNN embeddings. The evaluated resolutions were 2, 4, 8, and 16 levels, corresponding to 1-, 2-, 3-, and 4-bit representations, respectively. These conditions can be regarded as SLC-, MLC-, TLC-, and QLC-like signature representations. For a fair comparison, the same trained CNN, Omniglot test episodes, Gaussian random-projection matrix, 640-dimensional LSH signature, and 16-WL parity-split grouping were used for all quantization conditions. Each condition was evaluated using 600 paired test episodes so that the same support and query samples were compared across the different quantization resolutions.

Supplementary Fig. S2 shows the classification accuracy as a function of the quantization resolution for different $N$-way and $K$-shot settings. For the 5-way 1-shot task, the accuracies were 94.57%, 92.71%, 86.64%, and 73.02% for 2-, 4-, 8-, and 16-level quantization, respectively. For the 5-way 5-shot task, the corresponding accuracies were 98.68%, 98.21%, 96.18%, and 89.20%. In the more challenging 20-way condition, the 1-shot accuracies were 85.83%, 83.07%, 73.58%, and 54.73%, whereas the 5-shot accuracies were 95.73%, 94.88%, 90.72%, and 76.93%, respectively. These results indicate that the LSH-based FSL framework maintains high classification accuracy even with reduced quantization precision. In particular, the 2- and 4-level representations retain competitive accuracy, demonstrating that the FSL operation does not require high-resolution signatures to preserve the relative similarity among the support and query samples.

It should be noted that this quantization sweep was conducted using the sensing and similarity-scoring conditions established for the present TLC V-NAND implementation. The $V_{WL,CAM}$, parity-split search procedure, experimentally derived $I_{BL}$ lookup characteristics, and current-

based similarity metric were kept unchanged while only the number of quantization levels was varied. Therefore, the results quantify the sensitivity of the present TLCAM-based FSL framework to the LSH quantization resolution, rather than representing independently optimized SLC-, MLC-, TLC-, and QLC-based CAM systems.

When a different number of physical $V_{th}$ states is used, the PV spacing, search-voltage levels, $V_{WL,CAM}$, SA sampling conditions, quantization boundaries, and current-to-similarity mapping can be recalibrated for the corresponding memory mode. Accordingly, the accuracy reduction observed at 16 levels may originate not only from the increased quantization resolution, but also from applying TLC-optimized sensing and scoring conditions to a QLC-like signature representation without additional optimization. Memory-mode-specific hardware–software co-optimization may therefore further improve the classification accuracy at each bit resolution. Nevertheless, the present results demonstrate that the proposed LSH-based FSL scheme exhibits substantial tolerance to quantization resolution and retains high accuracy under low-bit representations.

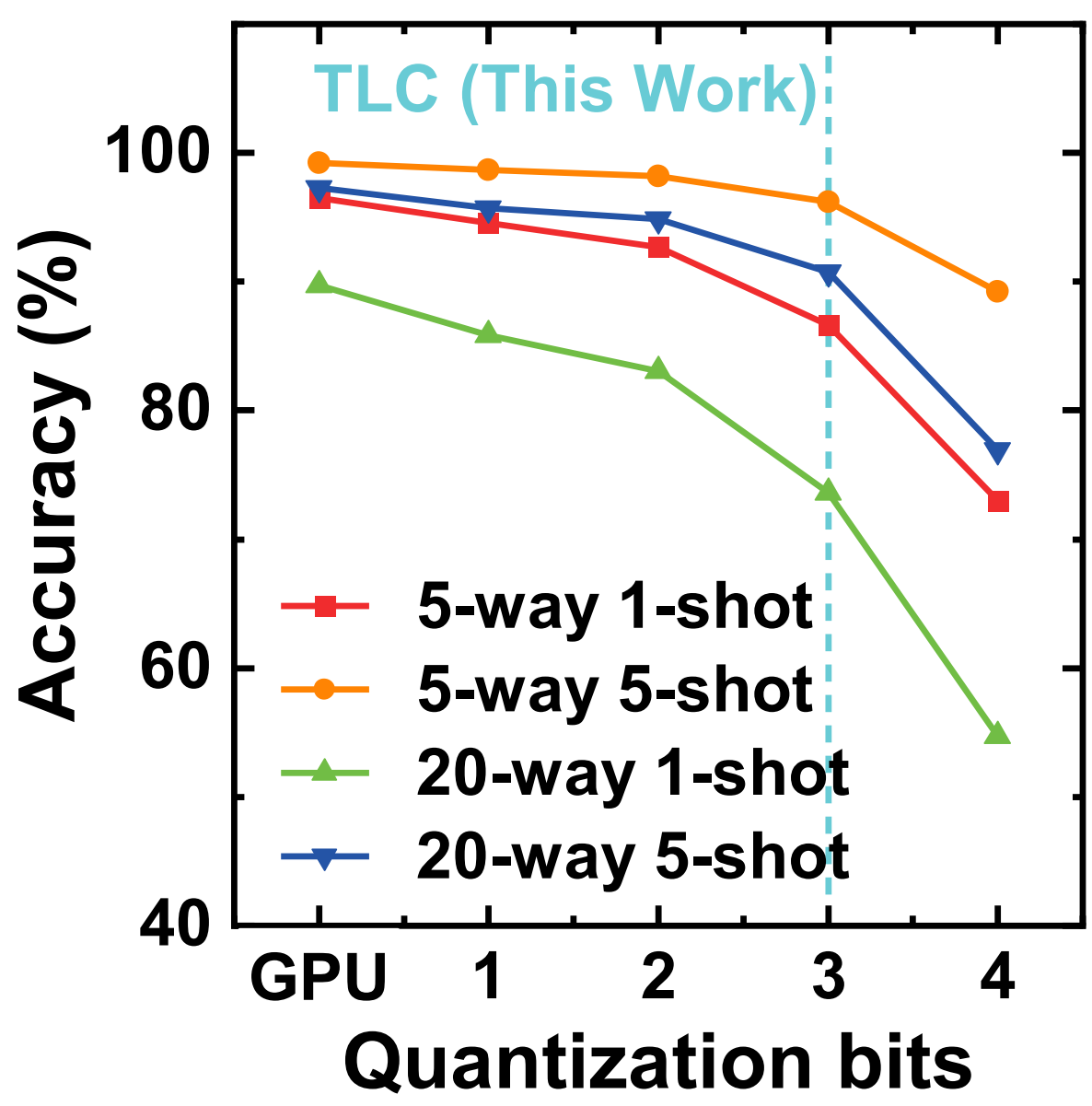


**Supplementary Fig. S2** Quantization-resolution sensitivity of the TLCAM-based FSL framework.
